# Physiological Responses to Organizational Stressors Among Police Managers

**DOI:** 10.1007/s10484-023-09613-2

**Published:** 2024-01-20

**Authors:** Paula M. Di Nota, Sarah C. Scott, Juha-Matti Huhta, Harri Gustafsberg, Judith P. Andersen

**Affiliations:** 1 Department of Psychology, University of Toronto Mississauga, Mississauga, ON Canada; 2 Police University College of Finland, Tampere, Finland; 3 Faculty of Culture & Education, Tampere University, Tampere, Finland

**Keywords:** Organizational stress, Occupational stress, Stress physiology, Heart rate, Stress management, Police training, Feasibility study

## Abstract

Police officers demonstrate increased risk of physical and mental health conditions due to repeated and prolonged exposure to stressful occupational conditions. Occupational stress is broken into two types: operational stress, related to the content of field duties (e.g., physical demands); and organizational stress, related to cultural and structural contexts (e.g., interpersonal relationships). Applied police research focuses on physiological activation in operational tasks as a mechanism explaining health risk and non-optimal performance outcomes. However, recent survey-based studies indicate numerous organizational stressors associated with self-reported mental health symptoms. The question of whether organizational stressors elicit significant physiological activity remains unknown. The current proof-of-concept field study tests the hypothesis that police managers will display significant physiological reactivity before, during, and after engaging in reality-based scenarios representative of stressful police management tasks developed from evidence-based pedagogical approaches. A sample of 25 training police managers (7 female, M = 16 +/- 5.3 years of experience) completed 5 reality-based scenarios, including resolving a heated conflict between colleagues, delivering negative feedback to a subordinate, and critical incident command. Significant increases in heart rate relative to rest were observed during all tasks, and in anticipation of several tasks. Greater increases in reactive heart rate were associated with longer recovery times. Sex differences and relationships between objective biological and subjective psychological measures of stress are discussed. The current findings demonstrate significant physiological responses to organizational stressors similar to levels observed during operational tasks, despite the absence of physical or aerobic exertion. Implications for police health and training are discussed.

**Supplementary Information:**

The online version contains supplementary material available at 10.1007/s10484-023-09613-2.

## Introduction

Police officers are at increased risk of physical and mental health conditions as a function of repeated and prolonged exposure to stressful occupational conditions (Violanti et al., 2017). Acute stress is associated with poor decision making, cognitive functioning and motor control (for review, see Anderson et al., 2019; Di Nota & Huhta, 2019). Chronic stress is associated with physical illness, dysregulation of normative diurnal cortisol patterns, and increased mental health symptoms in police and other public safety personnel (PSP) (Carleton et al., 2018, 2019a, b; Chan & Andersen, 2020; Planche et al., 2019; Sommer et al., 2020; Violanti et al., 2017).

According to established definitions, occupational stress (or stressors) can be divided into two distinct subtypes: operational and organizational. Operational stressors refer to the work-related content of one’s profession including physical conditions (i.e., environmental and equipment) and duties, shift work, overtime, and upholding a “higher image” to the public (CIPSRT, 2022; Ricciardelli et al., 2020). Operational stress injuries typically result from potentially psychologically traumatic events (PPTEs). PPTEs include real or threatened incidents that compromise physical, mental, or sexual wellbeing such as acts of violence or automobile accidents (Carleton et al., 2019a, b). In comparison, organizational stressors refer to the context within which professionals operate. Several recent reviews and survey-based studies have identified numerous organizational stressors, including interpersonal relations (e.g., a lack of social support between co-workers), toxic workplace culture, inconsistent leadership styles, and the bureaucracy of managing limited resources and infrastructure (Acquadro Maran et al., 2022; CIPSRT, 2022; Edgelow et al., 2022; Ricciardelli et al., 2020; Shane, 2010). Experimental studies that directly observe the relationships between stress, performance, and health in police have focused primarily on frontline officers in operational contexts both in the field (Anderson et al., 2002; Baldwin et al., 2019; Giessing et al., 2020) and reality-based scenarios (for review see Di Nota & Huhta, 2019). However, the extent to which organizational stressors elicit physiological reactivity, and its relationship to psychological measures of stress and health, remain unknown.

Police managers are an ideal population in which to investigate the physiological impact of organizational stressors. Within policing, managers are defined as sworn officers that assume authority over subordinates based on their hierarchical rank. Accordingly, their day-to-day duties can range from administrative tasks (e.g., scheduling, liaising with upper and lower ranks) to complex critical incident command (CIC) duties that reflect many of the organizational stressors identified above more so than operational stressors (Brown et al., 1996). Specifically, operational CIC tasks include oversight of decision-making and communication with police and other emergency services during time-sensitive, high-risk incidents (e.g., mass shooting, crowded protests). Taking responsibility during a CIC task is implicitly understood to be extremely stressful, given that they entail social repercussions and potentially life or death consequences for the persons that the police manager assigns to a particular operation. However, emerging research demonstrates that even relatively mundane organizational tasks, are reported to be a source of significant psychological stress among police and other PSP (e.g., firefighters, paramedics, Acquadro Maran et al., 2022; Chan & Andersen, 2020; Edgelow et al., 2022; Ricciardelli et al., 2020; Shane, 2010). Despite the growing focus on organizational stress, research examining the mechanisms by which police management tasks are associated with negative physical and mental outcomes is scarce.

Experimental research supports a purported mechanism by which organizational stress would be associated with physiological reactivity among police managers. Researchers demonstrate a link between negative social interactions and social exclusion to increases in cardiovascular metrics such as heart rate (HR) and heart rate variability (HRV) (Hidalgo-Muñoz et al., 2022; Liddell & Courtney, 2018). For example, Chen and Drummond (2008) showed that individuals show significant increases in HR, skin conductance and respiration during social evaluative tasks (i.e., singing in front of strangers, listening to audiotapes of themselves), regardless of whether they had a fear of social evaluation. Applying this literature to police managers, it is probable that organizational stressors will elicit physiological reactivity given that highly challenging tasks (e.g., managing subordinates, CIC) all entail social evaluation with major repercussions if mismanaged. Indeed, poor decisions and problematic CIC oversight can compromise public safety, as suggested in the aftermath of mass casualty events in Truro, Nova Scotia, Canada (2021) and Uvalde, Texas, USA (2022). Therefore, understanding the physiological mechanisms linking organizational stress to negative health outcomes is critical for both police and public safety, and builds on extant applied psychophysiological research in operational police contexts (see Table 1; Andersen et al., 2018; Arble et al., 2019; Baldwin et al., 2019; 2022; Chan et al., 2022; Giessing et al., 2020). Further, an examination of stress reactivity before and after individual tasks will provide novel insights into how anticipation, post-incident debrief and recovery are respectively related to psychological health and individual differences.

**Table 1:** Physiological Reactivity to Operational Stress in Police. The following table summarizes previously reported levels of physiological reactivity in police officers during scenarios or operational field duties. Sample descriptions include the sample size, type (i.e., frontline or tactical officers), and country. Task descriptions include whether physiological reactivity was measured during reality-based scenarios; virtual or video-based scenarios; or operational field incidents. Task type descriptors (e.g., critical incident scenarios, use of force scenarios) are derived from the original studies. Physiological reactivity is measured by heart rate (HR) reported in beats per minute (BPM) across all studies. The values reported in the final column are either average or maximum HR across one or multiple tasks that were considered high-threat (i.e., HR reported during low-threat scenarios are not included in the values listed below), and which occurred prior to any experimental intervention, training, or manipulation (i.e., only baseline or pre-test values of HR are included in the values listed below)

Author	Sample *n* Type (Country)	Task	Heart Rate (BPM)
Andersen & Gustafsberg, 2016	12 Federal Special Response Officers (Finland)	Reality-based scenarios (2)	160.63^*b*^
Andersen et al., 2015	24 tactical officers (17 in Finland; 7 in Canada)	Reality-based scenarios	148.8^b^
Andersen et al., 2016	24 tactical officers (17 in Finland; 7 in Canada)	Operational field incidents	146^b^
Andersen et al., 2018	57 police officers (Canada)	Reality-based scenarios (3)	115.68^b^
Anderson et al., 2002	76 police officers (Canada)	Operational field duties	82^*a*^
Arble et al., 2019	17 police cadets (Sweden)	Critical incident scenario	157.82^b^
Armstrong et al., 2014	41 police officers (Canada)	Use of force training scenarios (4)	116.19^a^
Arnetz et al., 2009	18 police officers (Sweden)	Critical incident scenario	120.12^a^
Baldwin et al., 2019	64 police officers (Canada)	Operational field duties	82.7^a^, 147.6^b^
Baldwin et al., 2022	122 police officers (Canada)	Lethal force scenario	128.98^a^, 152.5^b^
Kleygrewe et al., 2023	54 police officers (Netherlands)	Operational scenario (live)	88.63^a^, 136.96^b^
Kleygrewe et al., 2023	54 police officers (Netherlands)	Operational scenario (virtual reality)	90.91^a^, 126.28^b^
McCraty & Atkinson, 2012	59 police officers (United States)	Operational scenario real life; 3)	134.17^a^
Nieuwenhuys & Oudejans, 2010	7 police officers (Netherlands)	High threat scenario	122.5^b^
Nieuwenhuys & Oudejans, 2011	27 police officers (Netherlands)	High threat scenario	98.9^a^
Nieuwenhuys et al., 2012	36 police officers (Netherlands)	High threat scenario	97.01^a^
Nieuwenhuys et al., 2012	55 police officers (Netherlands)	High threat scenario	131.55^a^
Nieuwenhuys et al., 2015	16 police officers (Netherlands)	High threat scenario (virtual)	94.8^a^
Nieuwenhuys et al., 2015	13 police officers (Netherlands)	High threat scenario (real life)	105.8^a^
Oudejans, 2008	17 police officers (Netherlands)	High pressure scenario	116.1^a^

^*a*^ Average heart rate ^*b*^ Maximum heart rate

Based on the research gaps and priorities identified above, the current proof-of-concept observational field study tests the hypothesis that organizational stressors experienced by police managers will elicit significant physiological reactivity relative to rest. Evidence-based pedagogical approaches were used to develop reality-based scenarios tailored to reflect organizational stressors in representative police management situations. We predict that physiological reactivity will be observed before (i.e., anticipatory), during and after (i.e., debrief) the scenarios, even after accounting for individual differences (i.e., demographics, years of experience). We will also examine the persistence of physiological reactivity, as measured by recovery time from maximum to resting HR, following each task. Relationships between reactivity and self-reported ratings of psychosocial stress (i.e., operational and organizational stress, and mental health symptoms) will be examined.

## Methods

### Participants

A sample of 25 experienced police officers (7 female, *M*_*Age*_ = 40.7, *SD*_*Age*_ = 4.7, *M*_*Experience*_ = 16.0 years, *SD*_*Experience*_ = 5.3 years) were recruited from the Master of Police Services Program at the Police University College (PUC) in Tampere, Finland (Table 2). All participants were nearing the end of the Master’s program and had been in full-time studies (i.e., engaging in work-related training and educational activities and not in their traditional operational roles) for at least six weeks before the time of the current study^1^. All participants provided written informed consent and all procedures were approved by the University of Toronto Research Ethics Board, PUC, and adhered to the Declaration of Helsinki.

**Table 2:** Participant demographics and descriptive statistics

Variable	*M* (*SD*) range, *n*
*n* (Female)	25 (7)
Age	40.7 (4.7) 33–50, 25
Years of experience	16.0 (5.3) 6–27, 25
HADS-D	0.6 (0.96) 0–3, 25
HADS-A	1.9 (1.5) 0–6, 25
PCL-C	19.4 (2.6) 17–27, 25
GHQ-12	8.4 (2.1) 4–14, 25
PSQ-Op	2.19 (0.8) 1.0-4.3, 25
PSQ-Org	2.17 (0.7) 1.0-3.7, 25
HR Rest (in BPM)	
Day 1	76.78 (13.0) 58–102, 24
Day 4	85.80 (11.4) 65–103, 24
Day 5	83.63 (13.7) 64–111, 20
Day 6–8	91.34 (14.8) 69–118, 21
HR Max (in BPM)	
Computer-based CIC	84.6 (15.6) 54–124, 24
Promotion denied	119.1 (19.0) 80–150, 21
Late for meeting	119.9 (22.0) 66–147, 20
Principal task	98.6 (18.8) 64–128, 20
Live CIC	104.7 (19.6) 75–141, 20
HR Recovery (in sec)	
Computer-based CIC	549.7 (730.8) 0-2695, 23
Promotion denied	339.95 (527.2) 17-2294, 20
Late for meeting	211.8 (174.8) 40–749, 19
Principal task	38.1 (41.7) 4-174, 15
Live CIC	215.8 (303.7) 9-1089, 20

*Note*: Resting heart rate (HRRest) was measured over a 5-minute period of seated rest only on days when participants completed reality-based scenarios or tasks (see Table 3 for study schedule). For Days 6–8 HRRest was obtained from each participant immediately prior to completing the live critical incident command (CIC) task, which was completed individually on one of three days and part of participants’ educational curriculum

### Procedure

The current field study took place over two weeks at Finland’s PUC in March 2018. Prior to the start of the study, participants that agreed to take part completed a series of demographic and psychosocial questionnaires (described below). Participants completed five reality-based scenarios that were designed to assess physiological arousal as well as train and evaluate effective leadership, communication, and stress management skills. Over the course of the study, participants wore their uniforms (i.e., button shirts and trousers typical of police managers and leaders, not operational field uniforms) except for duty belts (i.e., no firearms), as they were engaging in classroom-based activities and preparing for office-bound roles. At the start of each study day, participants put on HR monitors to collect continuous physiological data that were later matched to researchers’ time-stamped notes to identify events of interest (see Measures section below). Table 3 summarizes the study schedule. Individual tasks and scenarios are described in detail in the next section.

**Table 3:** Schedule of educational curriculum and study evaluations

Day 1	Day 2	Day 3	Day 4	Day 5	Day 6–8
Computer-based CIC task	Psychoeducational lectures		*Morning*: Lectures *Afternoon*: ‘Promotion denied’ & ‘late for meeting’ scenarios	*Morning*: Lectures *Afternoon*: Principal task	Live CIC task

The morning of Day 1 introduced participants to the research team, goals of the research study, and schedule for the coming days. The afternoon comprised a computer-based CIC task. The following four days included psychoeducational classroom-based lectures and discussion on the following topics that are part of the Master’s program curriculum: the neurophysiological basis of stress; learning, evaluation, and performance under stress in policing; adaptive physiological stress management and coping skills; de-escalation and professional communication in police leadership contexts; mental imagery and visualization as effective learning strategies (see Andersen et al., 2021; Arpaia & Andersen, 2019; Di Nota et al., 2021c). On Day 4, participants were coached on applying their newly learned skills during two immersive, individual reality-based scenarios that were representative of what they would experience in their upcoming roles as leaders and managers. Participants were briefed prior to the start of each scenario and provided post-scenario feedback on their performance by police instructors and on their real-time physiological profiles by researchers that are experienced in delivering psychophysiological training to police across North America and Europe. On Day 5, the morning was spent on further psychoeducational content and “drills” of newly learned meta-cognitive skills. In the afternoon, participants completed a group-based principal task. The final day of the study involved completion of a live CIC task that was completed individually over the final three days of the study period.

Some participants missed individual tasks due to other work or family obligations, and the sample sizes of participants included in analyses are provided in text, figures, and tables where relevant. One participant agreed to discontinue participation due to lack of engagement and attendance (e.g., did not show up for sessions following Day 4).

### Evidence-Based Task Design & Descriptions

All the following tasks were designed for the purpose of the current observational study except for the final live CIC task, which was a mandatory component of the participants’ educational curriculum in the Master of Police Services program. Tasks were designed and delivered by experienced and qualified police instructors to reflect realistic, organizationally stressful police management situations. All PUC instructors, including those who designed the current study’s tasks, are required to complete educational course credits that provide an understanding of pedagogical theory and practice to promote learning in various trainee populations (e.g., novices, experienced officers, specialists) (Di Nota et al., 2021a). Therefore, instructors’ qualifications and expertise are not merely based on performance abilities and reputation (Staller & Körner, 2021).

Tasks were designed in accordance with evidence-based best practices for scenario design in policing (see Di Nota et al., 2021a; Jenkins et al., 2020). In accordance with Körner and Staller’s (2021) constraints-led pedagogical approach, the current tasks promoted functional integration of training content (i.e., professional leadership, interpersonal, and self-regulation skills) with the requirements and realities of the operational working environment for police managers. We designed a variety of representative tasks that had their own respective situational criteria (i.e., the ‘what’ dimension’). Individual participant responses (both behavioural and physiological) were considered during debriefing (i.e., ‘who’ dimension), which further personalized the learning experience. This non-linear training approach also emphasized that there is no single prescriptive or ‘ideal’ approach to any given situation, but rather acknowledged and supported multiple functional alternatives in achieving a desired outcome (Körner & Staller, 2021). Adhering to these pedagogical principles are suggested to promote learning and skill transfer from training to field settings in police (Körner et al., 2021). While the above cited works were published after the study period and focus on critical incident (i.e., use of force) contexts, evidence-based principles were applied to the development of scenarios that had a clear learning (or evaluation) objective: represent typical operational management and leadership encounters that reflect organizational stress as expressed by police (Shane, 2010). Tasks were designed to reflect organizational stressors but did not include potentially traumatic content that would undermine learning objectives, induce ‘training scars’, and increase the likelihood for future mistakes (Di Nota et al., 2021c; Jenkins et al., 2020; Leino et al., 2011).

*Computer-based CIC task –* Participants completed a 22-minute computer-based CIC task. The task was completed simultaneously by all participants in a classroom setting but each participant sat at their own computer and completed the task independently. Pre-recorded video and audio of incoming dispatch information on a simulated mass shooting, including the sounds of sirens from responding units, were displayed. The video was paused at regular intervals to prompt 7 decisions from the participants, including what further information they would request, any instructions for responding officers or units, and coordination with other units. Participants had 20-seconds to type their decisions before the video continued and presented new information (e.g., units en route, updates on students exiting the building, map of the building and location, suspect description).

*Promotion Denied –* Participants were individually seated at a desk awaiting the arrival of an actor that they were instructed to provide negative feedback to. Specifically, participants were told to deny the actor of a promotion that they had applied for. The actor was initially upset but maintained a normal demeanour and tone of voice. Then part way into the conversation, the actor stood up abruptly, slammed their hands down on the table, and began yelling and angrily gesturing at the participant, because they felt they deserved the promotion. The scenario ended shortly after the actor’s outburst, but participants were given a chance to de-escalate the situation. The actor was the same for all participants and was a PUC instructor experienced in acting in training scenarios.

*Late for Meeting* – The participant was told that they were in charge of leading a meeting with several people from the department under their command, and that the participant would arrive late to the meeting room. The participant was then reminded to use the adaptive stress management techniques at any time during the scenario and were provided a minute to reflect on these psychoeducational skills before beginning the scenario.

At the beginning of the scenario, the participant started down the hall towards the meeting room and heard a very heated argument going on between all the meeting participants. Actors were instructed to continue the disagreement while the participant entered the room and began to respond to the argument, but to ultimately comply with instructions from the participant, who was expected to break up the verbal fight, gain control of the situation, and de-escalate the meeting.

*Principal task –* Participants were randomly assigned to groups of 5, and were told they were going to complete a task at the same time but were not told any details about the task itself. Upon entering a meeting room, participants saw the Director of PUC (i.e., the Principal) seated on one side of a table along with 3 other members of the research team. Participants were seated across from the principal and were instructed that he would be asking each of them a pre-scripted question about their experiences with their training, the international research team, and about specific concepts they have been taught (e.g., “How will you apply the skills you have learned this past week in your future role as a police manager?”). One-by-one, participants were questioned and responded before their peers. Due to the organizational structure at the PUC in Finland, the Principal is highly regarded, and it is not common for students to interact with him. Once the last participant answered the principal’s question, the scenario was over and participants were led out of the room. After completing the task, participants were kept separate from the groups of officers that had not yet completed the task in order to maintain the element of surprise in facing the PUC Principal.

*Live CIC task* – This task was completed as a requirement in the PUC’s Master of Police Services curriculum. Participants were individually scheduled to complete this 45-minute task over the course of three days at the end of the study period. Participants met with the researchers 10 min before they entered the testing room to complete the task. They were fitted with the heart monitor and reminded to employ the stress regulation skills that they were taught over the course of the study. The participant then entered PUC’s CIC center, which featured a series of computer stations in front of several elevated television screens that presented various types of information, including maps of the incident location, a list of current active duty and responding officers, the front page of the local news website, a large timer displaying the minutes elapsed in the current task, and an information dispatch screen. Immediately prior to beginning the task, participants were given 5-minutes to gather and review information related to the scenario, which was scripted and timed to be exactly 30-minutes. A police instructor was seated in the command center with the participant, who also controlled the information being displayed on the screens as the scripted task proceeded. The researchers and two CIC employees were seated in a closed-off control room above the CIC center to record time-stamped notes and deliver the scripted dispatch information, respectively. The critical incident was a novel simulated active shooter situation and participants were responsible for processing incoming information, liaising with relevant units, departments, and services, and providing commands and instructions to responding units and providing a statement to the media. Participants that had completed their live CIC task were also allowed to observe others, but not speak or interfere with the ongoing task. Six participants had another participant in the room during their task, which was noted by the study team. At the end of the scenario, participants had a 10-minute debrief on their performance with the instructor. Due to the fact that the task was part of the graded Master’s curriculum, researchers were not permitted to listen to the debrief, nor were the authors given access to the performance ratings due to confidentiality reasons.

The above-mentioned tasks incorporated both organizational and operational stressors as identified in extant applied police literature (Acquadro Maran et al., 2022; Edgelow et al., 2022; Ricciardelli et al., 2020; Shane, 2010). Specifically, the computer-based and live CIC tasks involved critical and time-sensitive decision-making, scrutiny from peers, supervisors, and possibly the public; the ‘promotion denied’ and ‘late for meeting’ tasks reflected escalated interpersonal encounters that required professionalism and effective communication skills; the principal task involved performing in front of colleagues and superiors (i.e., hierarchical power dynamics), and possibly being subjected to judgment and social evaluation from peers (see Discussion on the role of social evaluative threat). The extent to which interpersonal relations, leadership styles, and workplace culture were regarded negatively in the current sample was not known but could be possible contributors to organizational stress.

### Measures

Participants completed a battery of pre-study measures as standard of practice in PSP studies (see supplementary materials for complete list). The psychosocial and physiological measures examined in the current study are listed below. Of note, performance ratings (i.e., scores for correct and incorrect responses or scaled ratings of how well participants did during the tasks) were *not* obtained, as the purpose of the current study was to develop scenarios on relevant organizational tasks and test if these tasks elicited significant physiological reactivity indicative of stress responses.

*Demographics.* Participants were asked to self-report sex, age, and years of experience. All participants were of the same race (i.e., Finnish white) and thus it was not considered as a covariate.

#### General Health Questionnaire 12 (GHQ12)

This brief survey measures mental distress and general health. This subjective self-report scale requires participants to respond to each question on a 4-point scale (not at all, no more than usual, rather more than usual, or much more than usual). The GHQ12 has been normed on large population samples of workers in North America and Europe (Lesage et al., 2011). Scale psychometrics include good content validity (Goldberg & Hillier, 1979), high internal consistency as measured by Cronbach’s alpha of 0.94, and high predictive validity with other tests of depression (Goldberg, 1985).

*Hospital Anxiety and Depression Scale (HADS)*. This 14-item ordinal scale was developed for physicians to determine levels of anxiety and depression among patients (Zigmond & Snaith, 1983). Scores can range between 0 and 21 for either anxiety or depression (7 items each). Although this test is not a definitive diagnosis of anxiety or depression, it is an indicator of such symptoms. Scale items for anxiety (HADS-A) has specificity of 0.78 and a sensitivity of 0.9. For depression, the HADS-D has a specificity of 0.79 and a sensitivity of 0.83. Bjelland et al. (2002) conducted a systematic review of many studies and identified a cut-off point of 8/21 for moderate to severe symptoms of anxiety or depression. Cronbach’s alpha for the HADS anxiety and depression subscales are 0.83 and 0.82, respectively (Bjelland et al., 2002).

*PTSD Checklist-Civilian (PCL-C)*. This 17-item checklist assesses symptoms of post-traumatic stress disorder (PTSD) such as “Repeated, disturbing memories, thoughts, or images of a stressful experience from the past?” rated on a 5-point Likert scale (not at all, a little bit, moderately, quite a bit, extremely). A cut off score of 50 indicates significant symptoms of distress (Blanchard et al., 1996). The PCL-C shows satisfactory internal consistency, with Cronbach’s alpha varying from 0.56 to 0.77 (Sveen et al., 2016).

*Police Stress Questionnaire (PSQ).* Developed by McCreary and Thompson (2006), the PSQ asks participants to rate items considered to be among the most stressful factors of police work. One subscale focuses on organizational stressors, including culture and organizational structure, while the other subscale focuses on operational stressors encountered in daily work. Items are rated on a 7-point Likert scale from “No stress at all” to “A lot of stress.” Organizational items (20) include “dealing with co-workers,” “staff shortages,” “too much computer work,” among other questions. Operational items (20) include “shiftwork,” “working alone at night,” “overtime demands,” and “risk of injury on the job.” PSQ norms and cut-off values, including consideration for norms reported by male and female police officers, are reported by McCreary et al. (2017). The PSQ shows good internal consistency, with a Cronbach’s alpha of 0.95 (Queirós et al., 2020).

#### Physiological Measures

As reflected by Table 1, HR is the most common biomarker of stress physiology in applied police research as it can be non-invasively measured using ambulatory HR monitors during occupational tasks. Further, HR is relatively more robust to respiration and movement artifacts that render other cardiovascular (e.g., HRV) and neuroendocrine measures (e.g., cortisol) unreliable during live-action events (Billman, 2013; Chan et al., 2022; Hayano & Yuda, 2019; Thayer & Sternberg, 2006).

Continuous HR was collected on all study days using Bodyguard 2 ambulatory monitors (FirstBeat Technologies Inc., Jyväskylä, FI) that were fixed to participants’ torsos with adhesive electrodes. HR was recorded at a rate of 1 Hz and de-identified data were uploaded to a remote server and analyzed offline. Resting HR (HRRest) was obtained at the start of each day and averaged across 5-minutes of seated rest (i.e., during classroom lecture or daily briefing) prior to any scenarios or tasks.

For each of the tasks described above, we identified the following physiological outcomes similar to those reported in previous studies (Andersen et al., 2018; Di Nota et al., 2021b): (1) average maximum HR (HRMax). HRMax was calculated by identifying the peak maximum HR value achieved during the task, and averaging this value with HR two seconds prior and two seconds following the peak HR. This was done to provide a more stable measure of maximal autonomic arousal and reduce the risk of outliers or aberrant readings. HRMax was identified during each task, the 10-seconds before the principal and live CIC tasks as measures of anticipatory stress, the 5-seconds before responding to the Principal in the principal task (i.e., response anticipation), and during the live CIC debrief (based on available time-stamped notes); (2) recovery time (in seconds) from peak HR in each task back to the HRRest value collected on the same day.

HRRest was missing in 11% of cases (11 missing values from a possible 100 available data points), HRMax was missing in 14.4% of cases (18 missing values from a possible 125 available data points), thus recovery time could only be computed in 107 possible cases. However, 14 of these (13%) did not return to resting heart rate before the HR monitor was removed that day.

### Statistical Analyses and Data Visualization

Descriptive statistics are provided in Table 2. To test whether HR was significantly greater than rest during each task, we compared HRRest to HRMax during anticipation, task, and debrief periods (as applicable) using repeated measures ANOVAs and pairwise comparisons. Sex and years of experience were included as covariates in all ANOVAs to explore their contributions to physiological outcomes but were not found to be significant. Significant sex- and age-related correlations are reported below. If normality assumptions were violated based on Shapiro Wilk tests, non-parametric equivalents were used. Effect sizes are reported where applicable using partial eta squared. Significance was set at *p* < 0.05 and adjusted for multiple comparisons with Bonferroni corrections where applicable. Only measures related to outcomes were included in the analyses.

HR data are visualized below based on the consistency of task start/stop times and durations between participants. For instance, the computer-based and live CIC tasks were precisely timed and were the same duration for all participants. However, the durations and start/stop times vary between participants for the ‘promotion denied’, ‘late for meeting’, and principal tasks (see task descriptions above for details) and are graphically represented accordingly using bar and line graphs, respectively. Relationships between psychosocial and physiological variables were also examined.

## Results

### Task-Related Physiological Activation

As reported below, all tasks elicited significant physiological activation relative to HRRest. Tasks performed on the same day (i.e., ‘promotion denied’, ‘late for meeting’), and components of the same task (i.e., anticipation, response, debriefing) demonstrated positively correlated HRMax values (ρs ≥ 0.588, *p* ≤ 0. 008).

#### Computer-Based CIC task

**Fig. 1:**
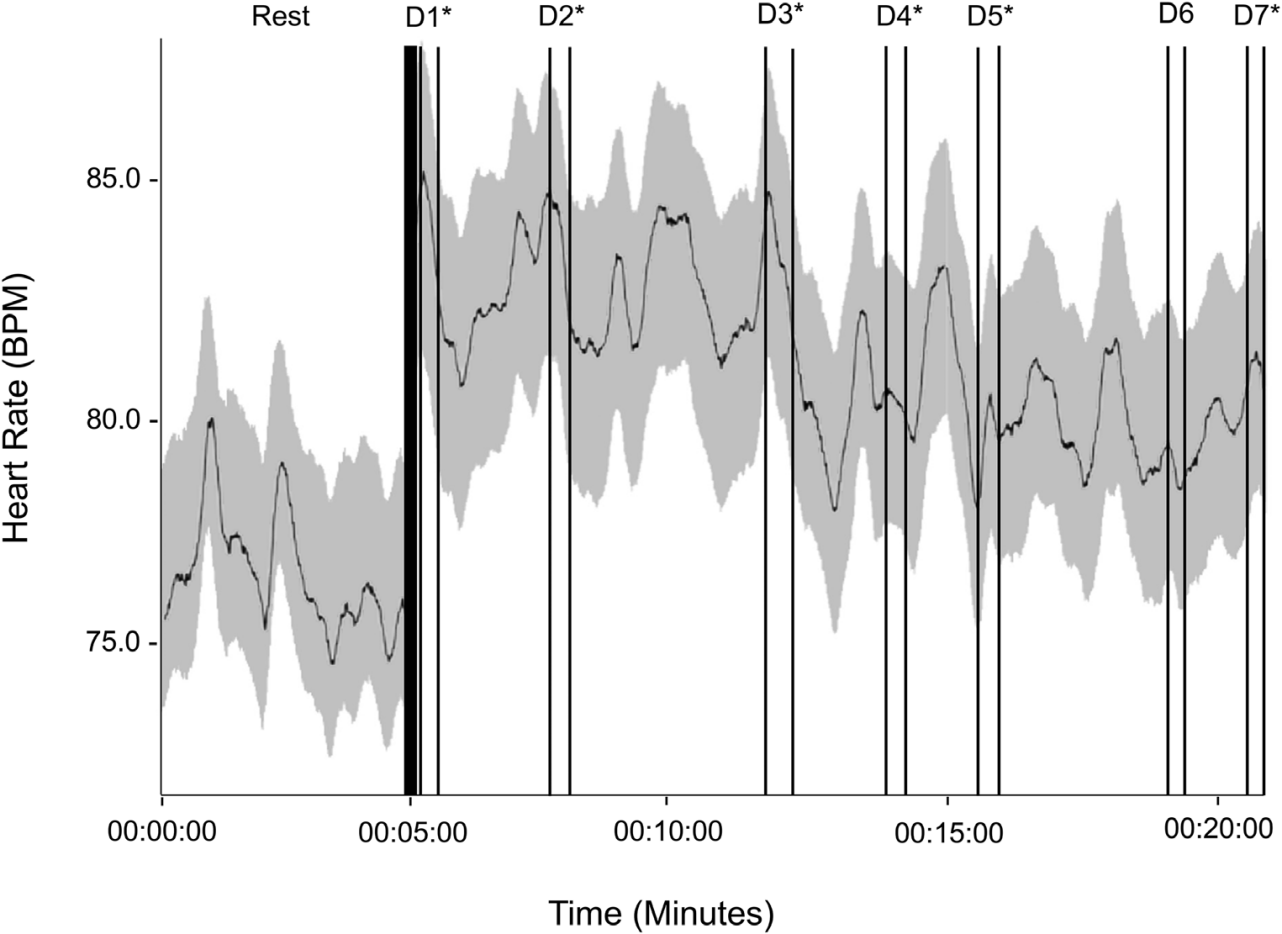
Physiological reactivity during a computer-based critical incident command (CIC) task. Continuous heart rate (HR, in beats per minute (BPM)) is plotted during 5-minutes of seated rest (left of the thick vertical line) and a 22-minute computer-based CIC task. Average HR values across 24 participants are shown by the black horizontal line, and one standard deviation is plotted in grayscale above and below the average HR line. During the task, participants watched a video of incoming dispatch information for a simulated active school shooter scenario. At regular intervals, the video was paused and participants had 20-seconds to type in a prompted decision. These seven decision points are indicated above with thin vertical lines and labels (D1 to D7). Average maximum HR was significantly elevated relative to rest at all decision points except for Decision 6 (D6). * *p*_*Bonf*_ < 0.05

A repeated measures ANOVA with a within-subject factor of time (8 levels: rest, 7 decision points) and between-subject factors of sex and years of service revealed that time was not a significant main effect (*F* (7,147) = 0.618, *p* = 0.741, η_p_^2^ = 0.029). Based on a priori hypotheses, pairwise comparisons revealed that all decision points except for decision 6 (*M*_*D1*_ = 85.84, *SD =* 17.6; *M*_*D2*_ = 86.13, *SD =* 16.0; *M*_*D3*_ = 85.43, *SD =* 16.0; *M*_*D4*_ = 83.10, *SD =* 15.3; *M*_*D5*_ = 83.96, *SD =* 15.6; *M*_*D6*_ = 82.43, *SD =* 16.0; *M*_*D7*_ = 82.67, *SD =* 15.4, *n =* 24 for all decision points) were significantly higher than HRRest (*M*_*HRRest*_ = 76.78, *SD* = 13.0, *n* = 24; *p*_*Bonf*_ < 0.05), and HRMax was not significantly different among the 7 decision points (*p*_*Bonf*_ > 0.05) (Fig. 1).

#### ‘Promotion Denied’ and ‘Late for Meeting’ Tasks

**Fig. 2:**
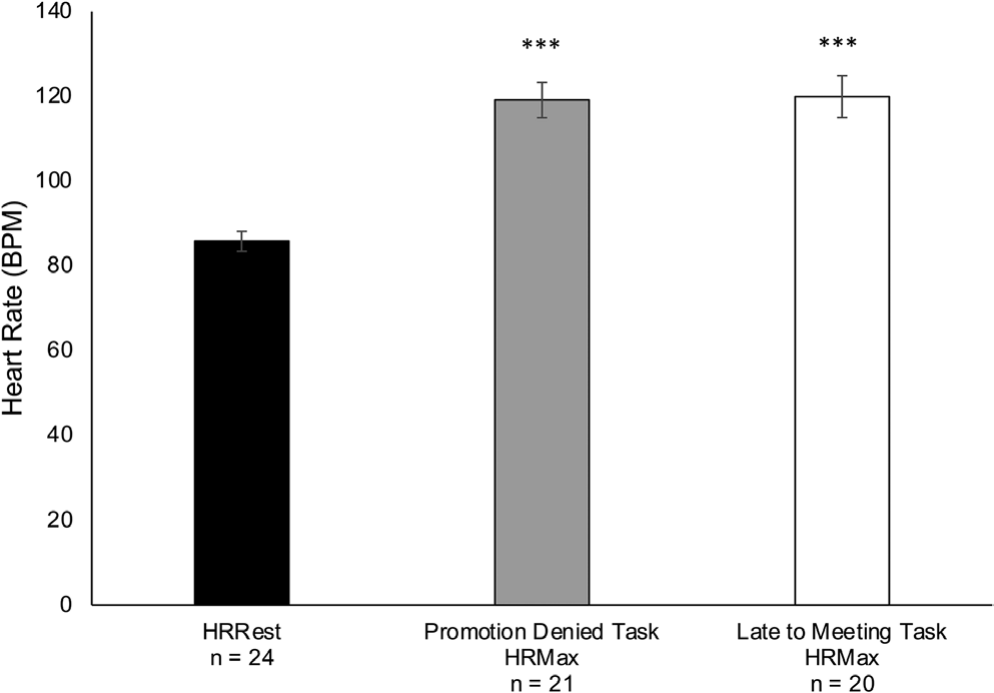
Resting and reactive heart rate (HR) during operational police management tasks. Average maximum HR (HRMax) was significantly higher during the ‘promotion denied’ (grey bar) and ‘late for meeting tasks’ (white bar) relative to rest (HRRest, black bar). Error bars show standard error of the mean. *** *p*_*Bonf*_ < 0.001

A repeated measures ANOVA with a within-subject factor of time (3 levels: HRRest; HRMax for ‘promotion denied’; HRMax for ‘late for meeting’) showed a non-significant main effect but moderate effect size (*F* (2,34) = 2.103, *p* = 0.138, η_p_^2^ = 0.110). Pairwise comparisons revealed that HRMax for both scenarios (promotion denied: *M =* 119.1, *SD =* 19.0, *n =* 21; late for meeting: *M =* 119.9, *SD =* 22.0, *n =* 20) were significantly higher than HRRest (*M =* 85.80, *SD =* 11.4, *n =* 24, *p*_*Bonf*_ < 0.001) (Fig. 2**)**. HRMax during the ‘late for meeting’ task was positively correlated to scores of mental distress on the GHQ-12 (ρ = 0.532, *p* = 0. 016, *n* = 20).

#### Principal Task

**Fig. 3:**
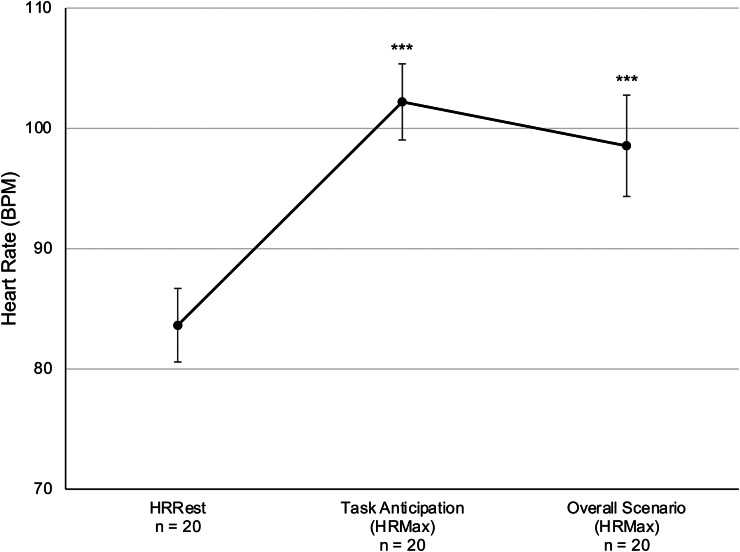
Resting and reactive heart rate (HR) during organizational police management principal task. Relative to rest (HRRest), average maximum HR (HRMax) was significantly elevated in anticipation of the principal task, prior to participants’ response to the Principal’s directed question, and during the highest peak HRMax during the overall task. Error bars show standard error of the mean. *** *p*_*Bonf*_ < 0.001

HRMax was not normally distributed for anticipation of the principal task, therefore a Friedman test for *k* repeated samples was applied to HRRest (*M =* 83.63, *SD =* 13.7, *n =* 20) and HRMax for task anticipation (*M =* 101.84, *SD =* 14.17, *n =* 20), response anticipation (*M =* 96.52, *SD =* 17.69, *n =* 20), and overall task reactivity (*M =* 98.28, *SD =* 18.7, *n =* 20) values. A significant difference was found between all time points (*χ*^2^(3) = 34.705, *p* < 0.001), and pairwise Wilcoxon Signed Ranks tests revealed that HRMax was significantly higher during all parts of the task compared to HRRest (*Z*s ≤ -3.582, *p*s < 0.001) (Fig. 3**)**, including anticipation of the overall task as well as during the 5-seconds prior to each participants’ response to the principal’s directed questions.

#### Live CIC Task

**Fig. 4:**
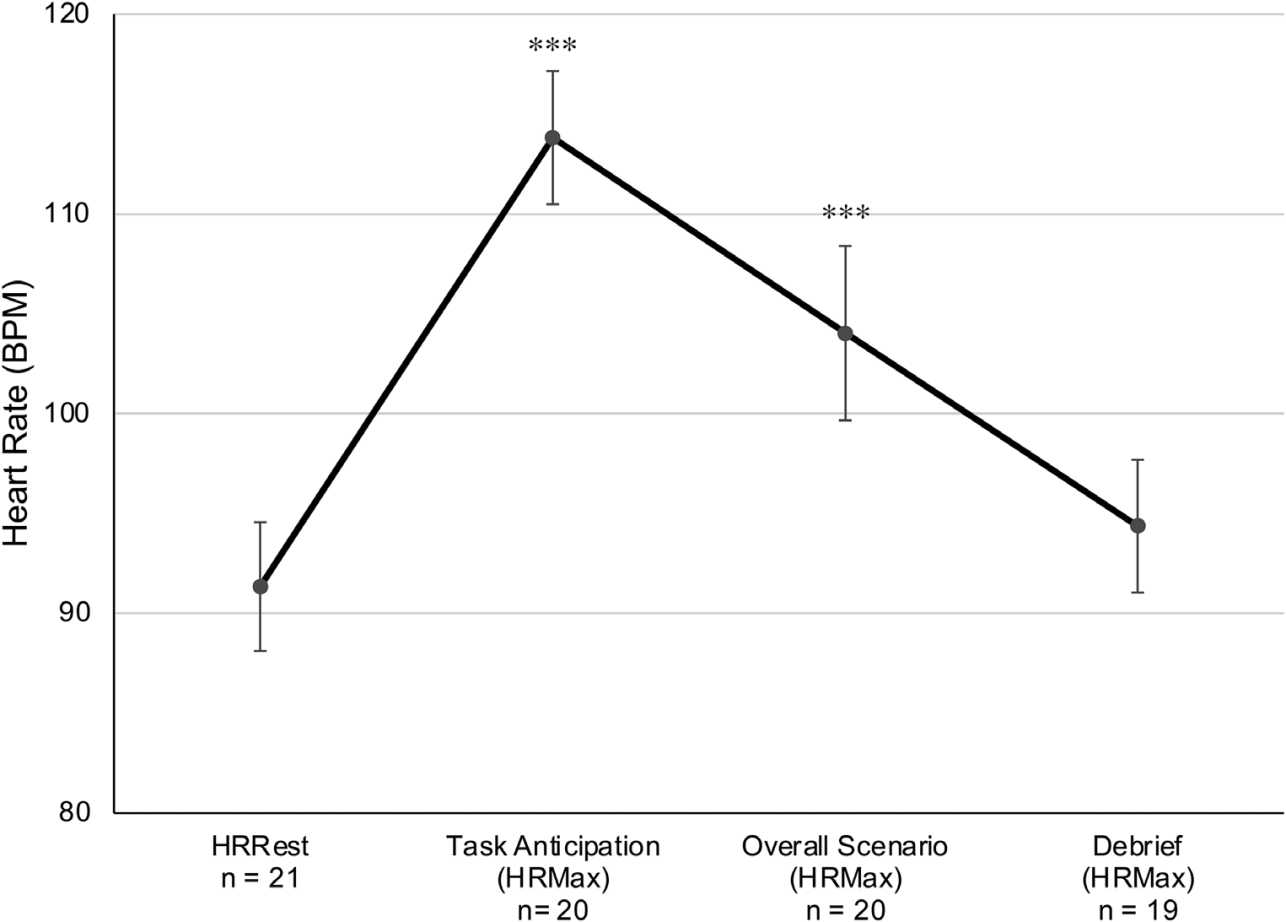
Physiological reactivity during a live critical incident command (CIC) task. Heart rate (HR, in beats per minute (BPM)) is plotted during 3-10-minutes of seated rest (HRRest), the peak HRMax achieved in the 5 min in anticipation of the CIC task, HRMax during the 30-minute task, and HRMax during the 10-15-minute post-task debrief period. Peak HRMax was significantly elevated in anticipation of and during the CIC task relative to rest, but not during debrief. *** *p*_*Bonf*_ < 0.001

**Fig. 5:**
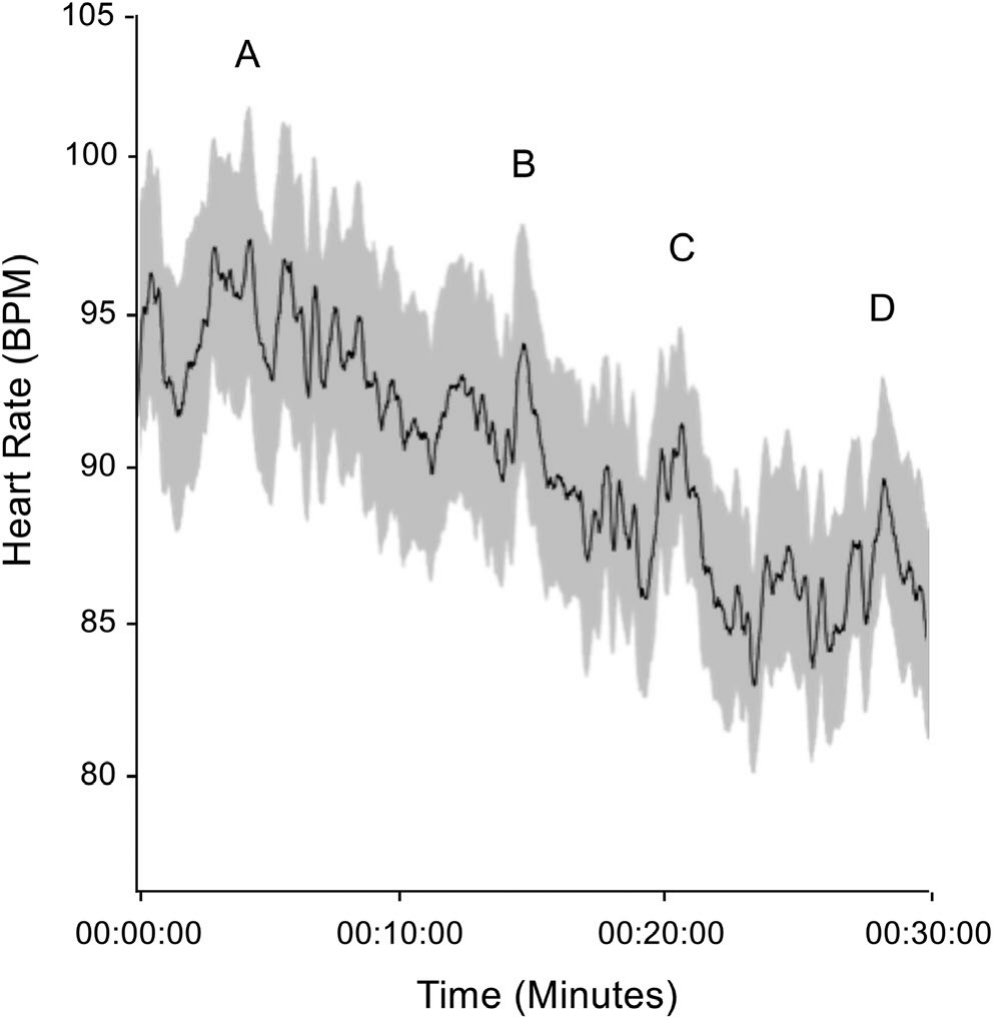
Physiological reactivity during a live critical incident command (CIC) task. Continuous heart rate (HR, in beats per minute (BPM)) is plotted for the 30-minute live critical incident command (CIC) task. Participants (*n* = 20) completed this task in accordance with their required Master of Police Services curriculum. Average HR values across participants are shown by the black horizontal line, and one standard deviation is plotted in grayscale above and below the average HR line. Clearly identifiable peaks in HR are marked with labels **(A-D)** and align with specifically scripted and timed task-related events. **A**: (5 min) police patrol shouts on the radio that the target person is raging and has a weapon, and that they want the commanding officer (i.e., the participant) to be aware of the situation (i.e., participant needs to take responsibility for the overall command and begin to make plans and operational decisions). **B**: (15 min) emergency dispatch states that there has been a call from a citizen reporting shots from a heavy calibre weapon fired near a public beach area. **C**: (21 min) police patrol states that they need to speak with the commander (participant) about a separate ongoing incident. **D**: (28-30 min) media has called the police dispatch asking for information about the ongoing shooting incident, and a separate news channel has already posted a story about what has happened, which the participant can see on one of the display screens

A repeated measures ANOVA with a within-subject effect of time (4 levels: HRRest and HRMax during anticipation, task, and debrief) with sex and years of service as covariates was not significant (*F* (3,48) = 0.476, *p* = 0.701, η_p_^2^ = 0.029). Based on *a priori* hypotheses regarding task-induced physiological arousal, we conducted pairwise comparisons that reveal significant reactivity relative to HRRest (*M =* 91.34, *SD =* 14.78, *n =* 21) during both task anticipation (*M =* 113.82, *SD =* 14.9, *n =* 20) and the CIC task itself (*M =* 104.03, *SD =* 19.5, *n =* 20, *p*_*Bonf*_ ≤ 0.001), but not for HRMax post-scenario debrief with an instructor (*M =* 94.37, *SD =* 14.5, *n =* 19, *p*_*Bonf*_ = 0.106, Fig. 4). HRMax was higher during task anticipation compared to peak HR during the task (*p*_*Bonf*_ = 0.040) and during debrief (*p*_*Bonf*_ < 0.001). Continuous HR was plotted for the duration of the task (Fig. 5) and reveals clear peaks in physiological activation that coincide with several pre-scripted events and prompts, including incoming dispatch reports of shots fired and requests for statements from the incident commander (i.e., participant) to attending officers and local news media stations. One-way ANOVAs reveal that HRMax did not differ between participants that had an observer in the room (*M* = 110.43, *SD* = 16.7, *n* = 6) and those that did not (*M* = 101.29, *SD* = 20.6, *n* = 14).

Prior to the final live CIC task, females self-reported significantly higher anticipatory stress (*U* = 4.50, *Z* = -2.821, *p* = 0.004) and expected higher difficulty on the upcoming CIC task relative to males (*U* = 11.50, *Z* = -2.014, *p* = 0.048). Participants who expected the live CIC task to be more difficult also self-reported higher anticipatory stress (ρ = 0.851, *p* < 0. 001, *n* = 17) and lower confidence in their decision-making following the task (ρ = -0.647, *p* = 0. 005, *n* = 17).

### Physiological Recovery from Organizational Stress Scenarios

As none of the recovery times were normally distributed (*p*s < 0.001), a Friedman test revealed significant differences between the five tasks (*χ*^2^(4) = 11.677, *p* = 0.020), with recovery time significantly faster following the principal task compared to the computer-based CIC, ‘promotion denied’ and ‘late for meeting’ tasks (*Z*s ≤ -2.355, *p*s ≤ 0.019, see Table 2 for descriptive summary).

There was a significant positive correlation between HRMax and recovery time on the ‘promotion denied’ (ρ = 0.695, *p* < 0. 001, *n* = 20), principal (ρ = 0.689, *p* = 0. 004, *n* = 15), and live CIC tasks (ρ = 0.497, *p* = 0. 026, *n* = 20), such that participants with higher HRMax took longer to recover. Females took longer to recover from the ‘late for meeting’ task relative to males (*U* = 15.00, *Z* = -2.105, *p* = 0.036).

### Resting HR Analyses

A repeated measures ANOVA with a within-subject effect of time (4 levels: HRRest on each of the four study days) was significant (*F*(3, 48) = 15.792, *p* < 0.001, η_p_^2^ = 0.497). As indicated by pairwise comparisons, HRRest on the final live CIC task day was significantly higher than HRRest on all other study days (*p*_*Bonf*_ ≤ 0.006, Table 2), and HRRest on Day 1 (computer-based CIC task) was significantly higher than HRRest on Day 4 (‘promotion denied’ and ‘late for meeting tasks’) (*p*_*Bonf*_ = 0.017).

Participants with higher HRRest on Day 4 had higher HRMax during the ‘promotion denied’ task (ρ = 0.471, *p* = 0. 031, *n* = 21). Similarly, participants with higher HRRest showed relatively less change from HRRest to HRMax when anticipating the principal (ρ = -0.468, *p* = 0.043, *n* = 19) and live CIC tasks (ρ = -0.559, *p* = 0.010, *n* = 20).

### Self-Reported Mental Health Symptoms & Demographics

Mental distress scores on the GHQ12 were positively related to symptoms of depression (ρ = 0.454, *p* = 0. 023, *n* = 25) on the HADS depression subscale. However, no other mental health scores were significantly correlated.

Females self-reported higher anxiety scores on the HADS compared to males (*U* = 28.5, *Z* = -2.141, *p* = 0.034) while older and more experienced officers reported higher anticipated difficulty of the computer-based CIC task (age: ρ = 0.647, *p* = 0. 005, *n* = 17; years of experience: ρ = 0.489, *p* = 0. 046, *n* = 17).

Consistent with established norms (McCreary et al., 2017), PSQ operational and organizational subscales were highly correlated (ρ = 0.635, *p* < 0.001, *n* = 25). Self-reported organizational stress (PSQ subscale) was not significantly correlated with HRMax before (anticipation), during (task max), or after (debrief) any of the tasks. However, the operational subscale of the PSQ was associated with greater increases in HRMax relative to rest during the computer-based CIC task (ρ = 0.581, *p* = 0.003, *n* = 24).

## Discussion

The current proof-of-concept observational field study found support for the hypothesis that police managers would display significant physiological reactivity relative to rest while engaging in organizationally stressful tasks. To our knowledge, physiological reactivity to organizational stressors has not previously been demonstrated in police management contexts and thus adds to the literature on applied psychophysiology and health among police. Specifically, officers demonstrated significant increases in HRMax in anticipation of and during all five reality-based scenarios. However, reactivity was not observed after the scenario (i.e., debrief), which was only measured following the live CIC task.

Resting HR on the final live CIC task day was significantly higher than HRRest on all other study days, and higher HRRest was significantly correlated with anticipation HRMax for the principal and live CIC tasks. Physiological recovery time following the tasks was highly variable between scenarios; individuals with higher HRMax took longer to recover than those with lower HRMax during three of the tasks (i.e., ‘promotion denied,’ principal, live CIC), and females took longer to recover from the ‘late for meeting’ task compared to males.

Age and years of experience were significantly correlated with each other, but not with other physiological or psychosocial scores. Similar findings were observed for the self-reported operational and organizational subscales of the PSQ, which were highly correlated with each other but not with physiological reactivity before, during or after any of the tasks. There were no significant associations between physiological reactivity and self-reported mental health symptoms. Females reported higher anxiety scores on the HADS compared to males, and also expected the live CIC task to be more stressful and difficult than males, according to self-reported ratings. Higher expected difficulty of the live CIC task was associated with lower confidence in their decision-making following the task.

The current findings underscore the need to examine and address occupational stressors more broadly beyond operational (e.g., use of force) police contexts in applied research and training, particularly considering the impact of sex and gender on psychological indicators of stress and health.

### Physiological Reactivity to Organizational Stress

The current findings align with contemporary experimental research and theory that demonstrates physiological reactivity is associated with aspects of organizational leadership and responsibilities that police managers are tasked with. Applied police research has most often measured physiological reactivity using cardiovascular autonomic metrics (i.e., HR, HRV) as summarized in Table 1. However, prior work with police has focused primarily on operational frontline duties, so we turn to insights from non-police populations to understand how organizational stressors may be related to physiological reactivity. For example, Iffland and colleagues (2014) review the characteristics of negative interpersonal interactions that are associated with increased HR. Specifically, the authors demonstrate that interactions containing the following are associated with distress and physiological reactivity: are potentially socially threatening; hold the possibility of social evaluation, rejection, or outright exclusion. Theory supports the association between organizational stress and physiological reactivity. Specifically, the Unease Modulation Model (UMM), an experiential model of stress, describes organizational defensive routines that increase physiological reactivity while reducing employee effectiveness (Arpaia & Andersen, 2019). Specifically, when any issue is emotionally charged, a person experiences an increase in unease, defined as the engagement of the autonomic nervous system via brain signaling. Unease can be measured peripherally with biomarkers including increases in HR and decreases in HRV. According to the UMM, the psychophysiological experience of unease hinders a person’s cognitive, communication and social skills, making it more difficult to complete tasks effectively, which further raises unease and physiological reactivity (Arpaia & Andersen, 2019).

Experimental research in non-police settings provides further evidence of biological mechanisms by which organizational stress may be linked to negative outcomes. Social Evaluative Threat (SET) refers to a situation in which one can be judged negatively by others. SET often occurs in the presence of others when persons are at risk of losing social status or acceptance. Previous work has found that SET resulted in increased cortisol, cardiovascular reactivity, and cardiovascular responses (Denson et al., 2012; Jordan & Smith, 2023; Woody et al., 2018). SET plays an important role in organizational stress, as organizational stress includes interpersonal communications and expectations (i.e., expectations from one’s supervisor). Given SET’s physiological impact on stress biomarkers, we find it unsurprising that organizationally stressful scenarios elicited significant increases in heart rate. SET may have played a particularly crucial role in physiological excitation in the principal task, as officers were performing in front of their highly respected Principal, as well as four of their peers. Perhaps the expectation to perform well in front of one’s peers specifically is in part responsible for the significant increases in anticipatory stress exhibited *before* entering the scenario room.

#### Physiological Reactivity and Recovery from Task-Related Anticipation and Post-Incident Debrief

A novel and unexpected finding was significantly higher physiological reactivity in the anticipation phase of the principal and live CIC tasks compared to HRMax during the tasks themselves (Figs. 3 and 4). Anticipatory stress can be adaptive by way of increasing an officers’ preparedness, vigilance, and attention. However, excessive levels of anticipatory stress can interfere with cognitive, emotional, verbal, and motor performance (Anderson et al., 2019; Arble et al., 2019; Di Nota & Huhta, 2019; Lehrer et al., 2020). Further, the UMM would posit that excessive anticipatory stress (i.e., unease) reduces the effectiveness of decision-making by prioritizing the reduction of unease over solving the problem (Arpaia & Andersen, 2019). Support for this relationship is provided by the significant correlation between self-reported difficulty in anticipation of the live CIC task and lower confidence in one’s decision-making following the task.

We also observed that resting HR was higher on the day participants completed the live CIC task. Furthermore, higher resting HR was positively correlated to higher HRMax during the principal and live CIC tasks, demonstrating a clear relationship between anticipatory stress as a driver of task-related reactivity. As the latter task was part of participants’ educational curriculum and bore more significant consequences than the other research-related tasks, relatively higher HRRest could reflect anticipatory stress of being in a position of increased accountability as a manager and incident commander. As an organizational stressor, a heightened sense of accountability for the consequences of one’s own actions has been related to increased anxiety, fear, and difficulty in police decision-making under stress (Verhage et al., 2018). Described in the UMM, feelings of uncertainty cause increases in unease (Arpaia & Andersen, 2019). CIC events are inherently uncertain, such that managers are required to make major decisions with little information available (Shortland et al., 2020). Consequently, CIC managers may choose not to change their course of action, even if new information reveals that a better operational or tactical strategy is available, simply because it reintroduces uncertainty that raises unease and resulting physiological reactivity (Arpaia & Andersen, 2019). Findings from a recent inquest on the CIC oversight during the mass casualty event in Truro, Nova Scotia, Canada supports this proposed link between uncertainty, unease and poor decision making (Shortland, 2022). Taken together, the prior research and current findings underscore the importance of recognizing internal physiological states as significant contributors to critical decision-making in stressful police management contexts.

The persistent effects of physiological reactivity are demonstrated by the significant positive correlation between higher HRMax and longer recovery time following three of the five police management tasks (‘promotion denied,’ principal, live CIC). The ability to recover from acutely stressful exposures in a timely manner has been identified as a hallmark of physiological resilience that reduces the likelihood of stress-inducing decrements to health and performance (Andersen et al., 2018; Arnetz et al., 2009). In the long-term, failure to recover physiologically can increase the risk for developing chronic physical and psychological conditions (see Sect. 4.2). Bertilsson and colleagues (2019) showed an accumulation of physiological stress responses (including HR) as multiple consecutive police tasks were performed, and which began during the anticipatory period. Accordingly, recovery is a critical physiological function for police that routinely face multiple calls in a single shift. In the current study, recovery time was highly variable between tasks and 13% of available cases (i.e., where resting and maximum HR values were obtained) did not return to HRRest before the end of physiological recordings. Just as occupationally mediated physiological reactivity *during* stressful tasks has been a focus of applied research, the current findings emphasize the importance of also examining its enduring effects beyond the immediate exposure (i.e., post-incident recovery).

Our limited analysis of physiological reactivity during debrief showed that HRMax was not significantly higher than rest following the live CIC task. All officers received detailed feedback and were questioned on their decision-making processes by instructors as part of their formal evaluation for this task. While the researchers did not have access to the instructors’ performance evaluations or observations of the participants during the debrief procedures, previous research suggests that the observed level of moderate physiological reactivity is conducive to reflection-based learning, which is beneficial in any training program (Di Nota et al., 2019, 2021a). Based on the extensive experience of the co-authors as police instructors in physiological stress management, tactics, and use of force (J-MH & HG), it is essential to gauge the learner’s current level of stress before conducting a debrief – and indeed prior to beginning a training scenario – to ensure that information is received and retained. Especially if performance errors have been made, instructors should attend to physical cues of heightened emotional and physiologically aroused states. Relevant physical cues include eye contact and gaze direction, posture and framing (i.e., turning away), and breathing rate and tone (i.e., shallow) (Chen & Drummond, 2008; Nieuwenhuys & Oudejans, 2011). Instructors should remediate physiological reactivity before any corrective feedback is given, including brief periods of paced breathing or prolonged exhalation, to bring learners to an optimal state where they will be receptive to learning and feedback. While basic science research shows that excessive physiological activation hinders memory encoding, a systematic review of police memory research shows limited insights regarding how stress reactivity before, during, and following critical incidents impacts their initial encoding, consolidation, and later recall, all of which remain ripe areas of future research (Di Nota et al., 2020b).

### A Physiological Mechanism for Negative Health and Performance Outcomes Due to Organizational Stress

We posit that chronic physiological reactivity elicited by occupational tasks is a central mechanism by which organizational stress leads to negative mental and physical health observed in police and other public safety personnel (Carleton et al., 2018; Violanti et al., 2017). This supposition is supported by decades of neurophysiological research demonstrating that sustained physiological reactivity during chronic stress rewires neural networks and negatively impacts metabolic, immune, endocrine and nervous system function, in addition to other system wide detriments (see Lovallo, 2016). Specifically, the theory of allostatic load demonstrates that sustained chronic stress places ‘wear and tear’ on the body, resulting in a reduction of physiological adaptivity to future stress and chronic physical health conditions (Schulkin, 2004).

The good news is that interventions that address maladaptive physiological responses to stress by teaching police to modulate their physiology demonstrate effectiveness above and beyond existing wellness programs (Andersen et al., 2023b; Di Nota et al., 2021a) For example, heart rate variability biofeedback (HRVBF) began to be used with law enforcement more than a decade ago (McCraty et al., 2009; McCraty & Atkinson, 2012). Advances in the use of HRVBF tailored to police specifically reveal immediate and sustained improvements in situational awareness, lethal force decision-making, and autonomic recovery from acute stress following a multi-day autonomic modulation intervention (Andersen et al., 2015, 2016, 2018; Andersen & Gustafsberg, 2016). The latter finding is especially relevant in police and PSP contexts where operators must recover quickly from one stressful incident to effectively manage the next call and avoid compounded physiological load (Baldwin et al., 2019; Bertilsson et al., 2019).

### Subjective vs. Objective Indicators of Stress: Associations among Psychosocial Factors

Despite the emphasis on organizational stressors presented in the current study tasks, scores on the organizational subscale of the PSQ were not significantly correlated with HRMax before (i.e., anticipation), during, or after any of the tasks, nor were they associated with any subjective task-related ratings of expected stress or difficulty. These findings are consistent with previous research showing discrepancies in subjective and objective measures of police attitudes and behaviour, reinforcing the need to reconsider how these factors are operationalized in research and practice (Andersen et al., 2023a; Di Nota et al., 2021b). McCreary et al. (2017) report normative PSQ values based on a large (*n* = 2840) sample of Canadian police officers as 3.26 (out of 7.0) for operational and 3.53 for organizational stress, while our study showed 2.19 and 2.17, respectively. While the current study sample reflects a similar proportion of male to female officers as the normative sample (70% and 80%), geographic and cultural differences may account for the relatively low PSQ scores observed presently (see Limitations section for further considerations on generalizability).

Self-reported measures of psychological distress and disorders were also well below established cutoffs for clinically significant symptoms of anxiety, depression, and PTSD (Table 2). There is substantial evidence to show that police are culturally stigmatized against seeking help for psychological stress and disorders (Carleton et al., 2019a; Ricciardelli, 2018). While SET related to mental health may account for the observed psychosocial scores, they may not have been underrated intentionally. Instead, experienced officers may habituate to a “new normal” and operate functionally under elevated levels of physiological and psychological stress, including significantly increased diurnal cortisol and rates of mental disorders relative to the general population (Carleton et al., 2018; Chan et al., 2022; Planche et al., 2019). The lack of significant association between any psychosocial (HADS, PCL-C, GHQ-12, PSQ) and physiological (HRRest, HRMax, recovery) outcomes in the current police sample supports this stress habituation theory.

#### Sex-Based Differences in Physiological and Psychological Stress

Our findings of higher self-reported anxiety in female participants compared to males is supported by previous empirical literature (Altemus et al. 2014; Donner & Lowry, 2013; Gater et al., 1998; Ranta et al., 2023). We also found that female officers rated the live CIC task would be significantly more “difficult” and “stressful” than male officers, which may be attributed to sex differences in self-efficacy. In general, Scandinavian women report lower self-efficacy than men (Bonsaksen et al., 2018), a result often found in educational research (Wang & Yu, 2023). As officers were graduate-level students at the PUC, reduced self-efficacy and confidence in one’s abilities among women in educational contexts may account for the higher ratings of anticipated difficulty and stress. While omnibus analyses of psychosocial and physiological outcomes in the total sample were non-significant, females took significantly longer to recover from HRMax to HRRest after the ‘late for meeting’ task. Increased anxiety and reduced self-efficacy may account for this finding, but there were no sex differences in recovery time following all other tasks (Table 2).

### Limitations

While the current sample is relatively small, our use of repeated measures design increase confidence in the ability to detect effects (Guo et al., 2013). A post-hoc examination of G*Power with the following parameters revealed sufficient power to detect effects: power = 0.76; effect size f = 0.25; α = 0.05; number of groups (within-subjects) = 1; repeated-measures (i.e., number of measurements for each scenario, which included anticipation, reactivity, and recovery) = 3. With respect to generalizability of the current findings to international police populations, we acknowledge several unique features of the study sample. Firstly, officers were enrolled in a Master of Police Services program at the PUC that qualifies them to serve as police managers and incident commanders. The professional training and educational requirements (i.e., both basic and advanced) of police officers in Finland and other European countries differs greatly from police organizations worldwide, such that training durations are typically much shorter and less comprehensive in North America (Belur et al., 2020; Di Nota & Huhta, 2019; Kleygrewe et al., 2022). In addition, we acknowledge the potential for cultural differences in the expression and experience of organizational stressors, including culture-specific norms of communicating with subordinate or superior members of one’s own organization (Brown et al., 1996). For instance, the Principal of the PUC does not often engage directly with students, which could account for the significant reactivity observed in anticipation of entering his office and responding to his direct questioning (Fig. 4). Finally, sociocultural differences in policing demands (e.g., crime rates, population characteristics, laws and regulations related to violent crime and weapon possession) and public perception may vary, as police officers are generally regarded highly in Finland (Vuorensyrjä & Fagerlund, 2018).

## Conclusions

The current findings further underscore the need to examine and address occupational stressors more broadly beyond operational (e.g., use of force) police contexts in applied research and training and consider the impact of sex and gender on psychological indicators of stress and health.

There is existing evidence showing that occupational stressors negatively impact the health and safety of police workers beyond the frontline, as civilian employees of Canadian police agencies report more suicide attempts that sworn officers (Di Nota et al., 2020a). Together with evidence from other public safety and frontline healthcare professionals, there is an urgent need to address the toxic organizational structures that have been shown to contribute to negative health outcomes (Birze et al., 2021; Ricciardelli et al., 2020). The good news is that researchers have pointed to the ways in which organizational stress is more controllable than operational exposures and may be dramatically improved from a top-down effort (Ricciardelli, 2018). Specifically, police organizations can change workplace structure, standards of communication, improve employee resources (e.g., staffing) and address toxic workplace culture. Prior research indicates that learning to modulate one’s own physiology during stressful tasks improves performance and health among police (Andersen et al., 2021). However, personal stress modulation alone is not enough to combat the effects of chronic organizational stress. The findings of the current paper can be used as evidence that organizational stress has real physiological implications and underscores the urgency for organizations to address their contribution to, and the resolution of, organizational stress to protect the health and longevity of their employees.

### Electronic Supplementary Material

Below is the link to the electronic supplementary material.

Supplementary Material 1

## Data Availability

The data that support the findings of this study are available from the corresponding author upon request.

## Footnotes

1 Participants alternated between regular full-time work duties during the previous summer and winter holiday periods before the study in March 2018, but timing of this information was not collected.
