# Supplementary material for: Physiological Responses to Organizational Stressors Among Police Managers
Source: Appl Psychophysiol Biofeedback. 2024 Jan 20;49(1):85–102. doi: 10.1007/s10484-023-09613-2 (PMC10869394; doi:10.1007/s10484-023-09613-2)
Supplement: Supplementary file 1 — Supplementary Material 1 [file 10484_2023_9613_MOESM1_ESM.pdf]

Supplementary Information

Article title: 'Anticipatory and Reactive Physiological Responses Among Police Managers to Organizational Stressors'
Journal name: Applied Psychophysiology and Biofeedback
Author names: Paula M. Di Nota[1], Sarah C. Scott[1], Juha-Matti Huhta[2,3], Harri Gustafsberg[2], & Judith P. Andersen[1]
[1] Department of Psychology, University of Toronto, Mississauga, Canada
[2] Police University College of Finland, Tampere, Finland
[3] Faculty of Culture & Education, Tampere University, Tampere, Finland
Email address of corresponding author: judith.andersen@utoronto.ca

Additional Psychosocial Measures

The following measures were included in the pre-study battery of questionnaires, but were not included in the main text due to a lack of significant relationships with primary outcomes of interest (i.e., physiological reactivity or recovery).

Alcohol Consumption. The AUDIT was developed by the World Health Organization (WHO) as a simple method of screening for excessive drinking and to assist in brief assessment. The scale consists of 10 items assessing frequency and amount of drinking. Items are rated on a Likert scale (never, less than monthly, monthly, weekly, daily, or almost daily). The second edition of the AUDIT was released in 1989 (WHO, 2001). The AUDIT has been used around the world as a valid and reliable measure of identifying problem trends in drinking habits.

Social Desirability: Items are selected from the Marlowe-Crowne (1960) Social Desirability Scale (MC). Items relevant to the construct of social desirability are included in order to assess the degree to which the officer is attempting to present an overly positive presentation of their personality. Items are rated True or False and include statements such as "I am always willing to admit when I make a mistake." "I am always courteous, even to people who are disagreeable."

State-Trait Anger Scale (STAS). The 10 item Trait-Anger portion of the STAS scale (Spielberger et al., 1983). This has been identified as a two factor scale "anger reaction" and "anger disposition." Items that represent reaction include "It makes me furious when I am criticized in front of other people." An item that represents anger disposition "I am a hotheaded person." A number of research studies have found an association between Trait-Anger and health outcomes such as blood pressure.

Brief Coping Orientation to Problems Experienced (COPE) Inventory. The 28 item COPE inventory serves to assess how people cope from a stressful life experience, including adaptive and maladaptive coping scales. Research on women with breast cancer has found the Brief COPE to be a reliable measure (Yusoff, Low & Yip, 2009). Items are rated on a 4 point scale from "I haven't been doing this at all" to "I have been doing this a lot" and include items such as "I've been expressing my negative feelings" and "I've been trying to find comfort in my religion or spiritual beliefs."

Citations:

Crowne, D. P., & Marlowe, D. (1960). A new scale of social desirability independent of psychopathology. *Journal of Consulting Psychology, 24*(4), 349.

Spielberger CD, Jacobs G, Russell S, Crane R (1983). Assessment of Anger: the State-Trait Anger Scale. In: Butcher JN, Spielberger CD, editors. *Advances in Personality Assessment.* Vol. 2. Hillsdale, NJ: Erlbaum

World Health Organization, Babor, Thomas F., Higgins-Biddle, John C., Saunders, John B. & Monteiro, Maristela G. (2001). AUDIT: the alcohol use disorders identification test : guidelines for use in primary health care, 2nd ed. World Health Organization. https://apps.who.int/iris/handle/10665/67205

Yusoff, N., Low, W. Y., & Yip, C. H. (2010). Reliability and validity of the Brief COPE Scale (English version) among women with breast cancer undergoing treatment of adjuvant chemotherapy: a Malaysian study. *The Medical journal of Malaysia*, *65*(1), 41–44.
